# Evaluation of a Health Information Technology–Enabled Collective Intelligence Platform to Improve Diagnosis in Primary Care and Urgent Care Settings: Protocol for a Pragmatic Randomized Controlled Trial

**DOI:** 10.2196/13151

**Published:** 2019-08-06

**Authors:** Valy Fontil, Elaine C Khoong, Mekhala Hoskote, Kate Radcliffe, Neda Ratanawongsa, Courtney Rees Lyles, Urmimala Sarkar

**Affiliations:** 1 Division of General Internal Medicine University of California San Francisco San Francisco, CA United States; 2 UCSF Center for Vulnerable Populations University of California San Francisco San Francisco, CA United States

**Keywords:** decision support systems, clinical diagnosis, medical informatics

## Abstract

**Background:**

Diagnostic error in ambulatory care, a frequent cause of preventable harm, may be mitigated using the collective intelligence of multiple clinicians. The National Academy of Medicine has identified enhanced clinician collaboration and digital tools as a means to improve the diagnostic process.

**Objective:**

This study aims to assess the efficacy of a collective intelligence output to improve diagnostic confidence and accuracy in ambulatory care cases (from primary care and urgent care clinic visits) with diagnostic uncertainty.

**Methods:**

This is a pragmatic randomized controlled trial of using collective intelligence in cases with diagnostic uncertainty from clinicians at primary care and urgent care clinics in 2 health care systems in San Francisco. Real-life cases, identified for having an element of diagnostic uncertainty, will be entered into a collective intelligence digital platform to acquire collective intelligence from at least 5 clinician *contributors* on the platform. Cases will be randomized to an intervention group (where clinicians will view the collective intelligence output) or control (where clinicians will not view the collective intelligence output). Clinicians will complete a postvisit questionnaire that assesses their diagnostic confidence for each case; in the intervention cases, clinicians will complete the questionnaire after reviewing the collective intelligence output for the case. Using logistic regression accounting for clinician clustering, we will compare the primary outcome of diagnostic confidence and the secondary outcome of *time with diagnosis* (the time it takes for a clinician to reach a diagnosis), for intervention versus control cases. We will also assess the usability and satisfaction with the digital tool using measures adapted from the Technology Acceptance Model and Net Promoter Score.

**Results:**

We have recruited 32 out of our recruitment goal of 33 participants. This study is funded until May 2020 and is approved by the University of California San Francisco Institutional Review Board until January 2020. We have completed data collection as of June 2019 and will complete our proposed analysis by December 2019.

**Conclusions:**

This study will determine if the use of a digital platform for collective intelligence is acceptable, useful, and efficacious in improving diagnostic confidence and accuracy in outpatient cases with diagnostic uncertainty. If shown to be valuable in improving clinicians’ diagnostic process, this type of digital tool may be one of the first innovations used for reducing diagnostic errors in outpatient care. The findings of this study may provide a path forward for improving the diagnostic process.

**International Registered Report Identifier (IRRID):**

DERR1-10.2196/13151

## Introduction

### Background

Diagnostic errors (defined as missed, delayed, or wrong diagnoses) in primary care affect an estimated 1 in 20 US adults every year [1]. About half of these errors can lead to serious preventable harm, but few interventions have been developed and tested to reduce diagnostic errors in real-world ambulatory care settings such as primary care or urgent care clinics [1-4]. This significant gap in clinical practice carries tremendous public health implications. Most individuals receive care in ambulatory care settings [5]. Diagnosis is particularly challenging in primary and urgent care because of 2 structural factors: (1) the short time duration and pressure to complete visits affects cognition and (2) primary care encompasses the broadest range of clinical concerns, from common diseases to rare conditions [6].

In current usual practice, clinicians at primary care and urgent care clinics commonly diagnose patients independently without collaboration or consultation with other health professionals or the use of health information technology (IT), potentially leading to an increased risk of diagnostic errors [7]. In focus groups about outpatient diagnosis, clinicians identified the use of technology to improve communication among them as a key strategy to enhance timely and accurate diagnosis [8]. In its recent report on diagnostic error, the National Academy of Medicine suggested that health systems employ 2 key strategies that are essential to reducing diagnostic error in the ambulatory care setting: (1) enhance interclinician collaboration and (2) develop and utilize health IT innovations in the diagnostic process [6]. A collective intelligence technology platform could be a promising tool to implement these 2 key strategies.

Collective intelligence is defined as shared or group intelligence that emerges from the collaboration or collective efforts of many individuals. It harnesses the ability of a group to outperform the individual in a variety of cognitive tasks [7]. IT platforms offer the opportunity to connect people and harness their collective intelligence through crowdsourcing—the practice of obtaining input into a task or project by enlisting the services of a large number of people via the internet [9]. In discrete tasks related to medical decision making, such as classification of radiology scans and pathological specimens, collective intelligence technology has been shown to improve accuracy when compared with individual decision making [10,11]. However, the impact of collective intelligence technology on diagnostic accuracy remains unproven in outpatient practice. Studies examining collective intelligence technologies have shown that users are enthusiastic about cross-discipline collaboration and easily obtaining expert feedback but wary of inaccuracies and inefficiency of using a collective intelligence tool [12].

In previous simulation testing, the collectively derived output from a collective intelligence platform outperformed its individual physicians in identifying the correct diagnosis in its assessment of standardized clinical cases [13,14]. However, effective implementation of such a tool in the outpatient setting requires examination of its efficacy and usability in real-world primary care and urgent care clinics.

### Study Objectives

In this pragmatic randomized controlled trial, we will examine the efficacy of a collective intelligence technology platform on improving primary clinicians’ confidence in their diagnostic assessments and their accuracy in making a correct diagnosis. We will also examine clinicians’ perceptions of the usability of the collective intelligence technology platform and their likelihood of using (ie *,* intention to use) such a platform in routine primary care or urgent care practice to assist with the diagnostic process.

### Ethical Approval

This study was reviewed and approved by the institutional review board at the University of California, San Francisco.

## Methods

### Setting and Study Population

We will use a convenience sampling approach to recruit primary care and urgent care clinicians in San Francisco, including clinicians from University of California San Francisco (UCSF) primary care clinics and San Francisco Department of Public Health (SFDPH) health care system, which includes 12 urban safety-net primary care clinics that serve a low-income and racially and ethnically diverse patient population. This system is an integrated care system with primary care clinics throughout the city of San Francisco, including 2 academic clinics that are staffed by UCSF faculty physicians. The clinics also provide urgent care through clinic sessions (half days or entire days) reserved for urgent care visits.

### Intervention

The intervention in this study is the provision of a Web-based collective intelligence output to primary care clinicians within 80 hours of a case presentation to assist them in the diagnostic process for routine clinical cases that do not yet have an established, confirmed diagnosis.

### Collective Intelligence Platform

**Figure 1:**
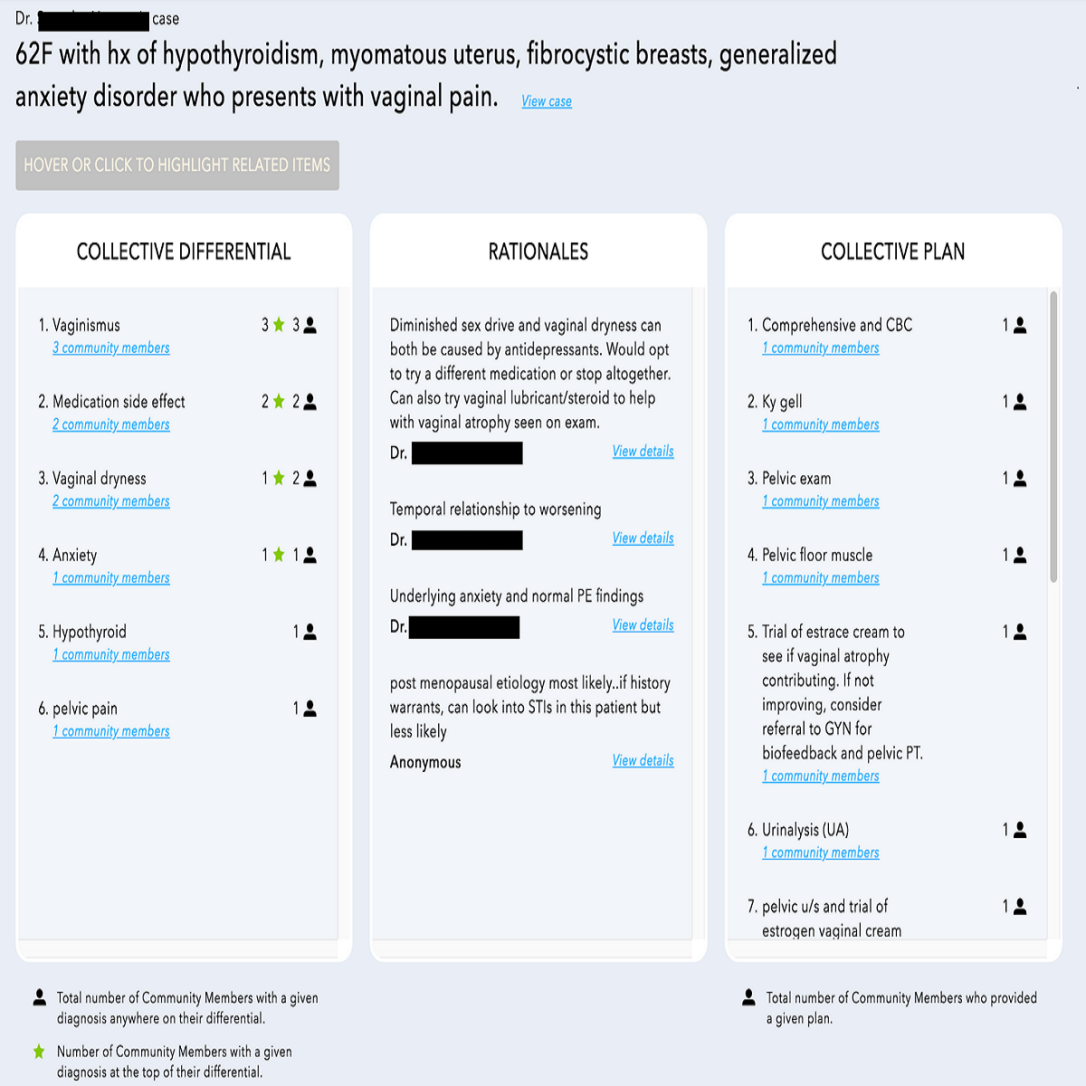
Screenshot of a collective intelligence output from the Human Dx platform for a clinical case. The columns display the collective intelligence output with the differential diagnosis, plan, and rationales. The interface is interactive – users can hover or click to see details of the case that was entered and details on the output differential diagnosis, rationales, and plan.

The Human Diagnosis Project (Human Dx) is a Web-based and mobile collective intelligence platform designed to implement both key strategies for reducing diagnostic error recommended by the National Academy of Medicine—interclinician collaboration and use of health IT in the diagnostic process—by utilizing collective intelligence among clinicians. Clinicians input the relevant details of a clinical case or question into the Human Dx platform. Afterward, any number of clinicians (including peers and specialists) participating on the platform, a minimum of 5 for this study, independently review the case and provide their own differential diagnoses and management plans. Using advanced techniques including prefixed search, autocomplete, and natural language understanding, Human Dx is able to structure clinicians’ clinical assessments and aggregate them to produce collective intelligence [13]. The collective intelligence output consists of (1) a collective differential diagnosis derived from a synthesis of respondents’ differential diagnoses, (2) a collective management plan derived from a synthesis of respondents' management plans, and (3) free-text explanations from respondents of the rationales behind their differential diagnoses and plans (Figure 1). Human Dx has been available since 2014 and has over 21,000 physician and medical student users who have solved or entered at least one case. To date, there are over 282,000 entered or solved cases on the platform.

### Study Design

**Figure 2:**
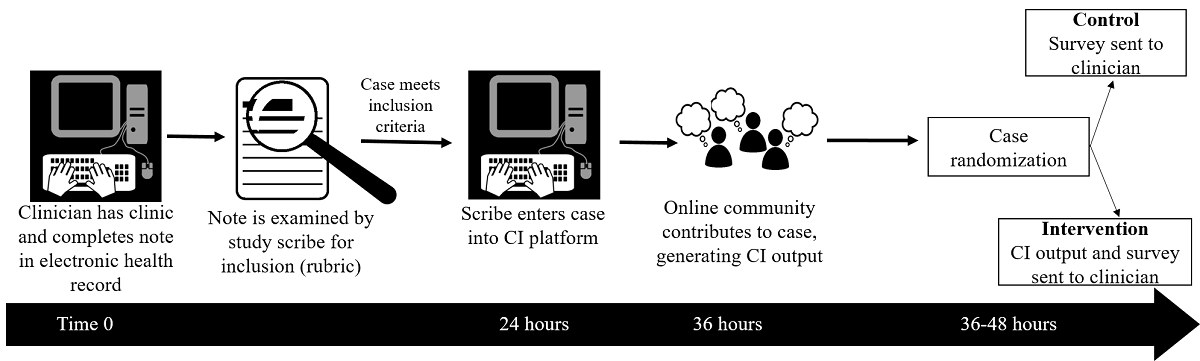
Workflow for participants. At baseline clinicians complete their normally-scheduled clinical sessions and complete their notes in the electronic health record. A study scribe examines each note and determines inclusion based on a study rubric. The study scribe enters the case into the collective intelligence (CI) platform within 24 hours and online contributors provide their own assessments to generate a CI within 36 hours. The CI output is sent to the study team, who randomize each case, and send the CI output to participants for cases randomized to the intervention. Participants receive a post visit questionnaire for all study cases (intervention and control).

This is a pragmatic randomized controlled trial in which primary care clinicians are followed over 4 to 8 weeks to identify a minimum of 20 cases, for each clinician, with potential diagnostic uncertainty. Half of the selected cases for each clinician will be randomized to the intervention and the clinician will receive a collective intelligence output for those cases. Human Dx will generate collective intelligence for the remaining (control) cases, but clinicians will not receive the collective intelligence output for these cases (Figure 2).

### Selection, Entry, and Randomization of Clinical Cases

For the purpose of this study, clinicians will not enter their own cases. For case entry, we will utilize study scribes who are medical doctors (MD) in their second or third year of internal medicine residency at UCSF. These scribes are analogous to intended real-world users of the Human Dx platform (ie, primary care and urgent care medical providers). On the basis of each participant’s clinical schedule, study scribes will access the electronic health record within 24 to 36 hours after the participant’s clinic session and identify cases that meet the study selection criteria (see below).

Study scribes enter cases that meet the inclusion criteria into the collective intelligence platform. A minimum of 5 clinicians will act as *contributors* who review and comment on each case in the collective intelligence platform within 24 to 36 hours of case entry, generating a collective intelligence output. The contributors recruited by Human Dx for this study were US-based attending-level physicians in internal medicine or family medicine. They were recruited from an existing pool of active Human Dx users (ie, they had solved at least one case within 3 months of being recruited for the study), which does not include any of the clinician participants in our study population. They are not affiliated with our study team and are blinded to our study procedures including case randomization (Figure 2).

Once the collective intelligence output becomes available for a case, the study team randomizes it to the intervention or control cases. The study team assigned each case a unique study identification number for tracking purposes. Using a random number generator in blocks of four, we will randomize half of the cases, for each clinician, to an intervention group in which the clinician will receive a collective intelligence output between 60 and 80 hours from the time the clinician saw the patient in the clinic (ie, within 24-36 hours of case entry). For cases randomized to the controlled group, Human Dx will generate collective intelligence, but it will not be shared with the clinician. Clinicians will complete an online postvisit questionnaire for all of their cases (control and intervention) that provides their initial impressions of the case (eg, differential diagnosis and perceived case difficulty) and level of diagnostic confidence. The questionnaire for cases assigned to the intervention group includes additional questions that the clinician will answer after reviewing the collective intelligence output to assess the effect of the output on their diagnosis, plan, and confidence. The study scribes and the *contributors* on Human Dx will be blinded to case randomization.

### Case Selection Criteria

Scribes will select cases based on a rubric designed to capture diagnostic uncertainty. If a case meets any of the following criteria, it will be entered into the Human Dx platform: (1) a new symptom or abnormality in laboratory, physical exam, or radiographic finding; (2) a recurrent symptom or abnormality in laboratory, physical exam, or radiographic finding without a clear diagnosis or etiology; (3) a pending diagnostic test ordered to evaluate a new or unresolved condition; (4) empiric treatment provided without complete diagnostic certainty; and (5) if the clinician submitted a request for electronic consultation from a specialist within the health network for a diagnostic or management problem. Clinicians may also flag a case for review that the scribes will enter into the Human Dx platform whether or not it meets the above rubric’s selection criteria.

### Data Collection and Study Procedures

#### Study Period

**Figure 3:**
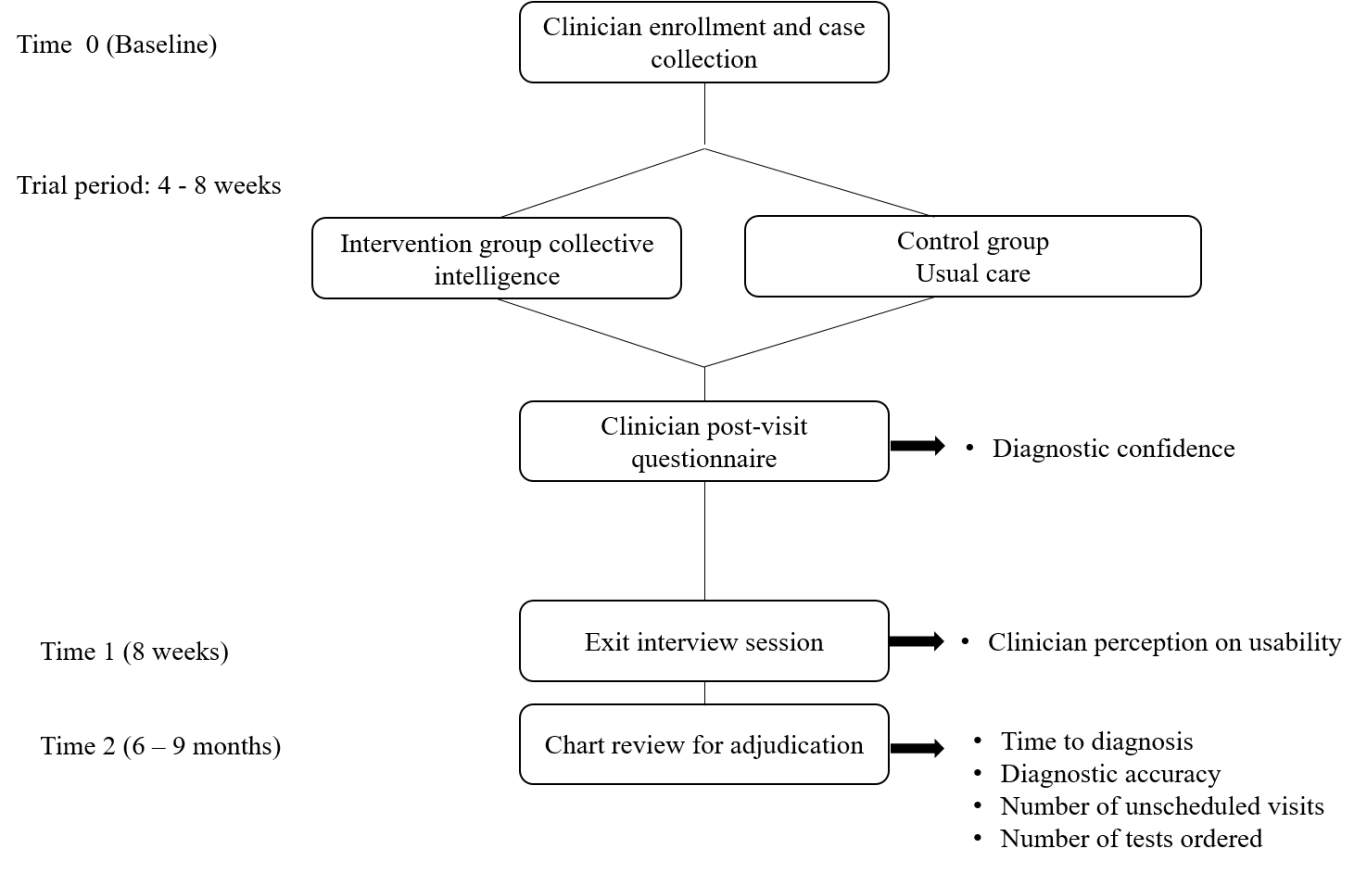
Study procedures. At baseline, clinicians are recruited and cases are collected and randomized to the intervention and control groups. Post-visit questionnaires will assess diagnostic confidence (the primary outcome) for each case. Exit interview sessions at 8 weeks will explore clinicians’ perception on usability. At 6-9 months, will conduct electronic medical record review to adjudicate clinic outcomes (secondary and exploratory outcomes).

We will follow participants’ consecutive clinic sessions over a period of 4 to 8 weeks to select a minimum of 20 cases per clinician. After all postvisit questionnaires are completed, the participant will participate in a 1-hour exit interview including an in-person survey and semistructured interview to assess the efficacy and usability of collective intelligence for medical diagnosis from the perspective of primary care and urgent care clinicians (Figure 3).

#### Postvisit Questionnaires

For cases randomized to the control group, a research assistant will email the study participant a brief one-line description of the case accompanied with instructions to fill an online postvisit questionnaire (Multimedia Appendix 1) that captures the participant’s clinical thinking. The brief one-line description is important to alert the participant to which case the questionnaire is referring; we will keep each one-line description in the study’s records to act as a case identifier. For cases randomized to the intervention, the participants will receive instructions to complete an online questionnaire in similar fashion as described above for the control cases. In addition, we will provide the participant a Web link to access the collective intelligence output for the intervention case as well as the questionnaire that will assess the impact of the collective intelligence on their clinical thinking (eg, confidence and decision making; see Multimedia Appendix 1). All instructions to participants will be sent as a single email. Participants will be instructed in the email to review the collective intelligence output for intervention cases only when prompted, to ensure that they respond to survey questions designed to assess their initial clinical thinking (for both intervention and control cases) before reviewing the collective intelligence output that will be available only for intervention cases.

#### Exit Survey and Interview

We will conduct an exit session to complete an in-person exit survey and semistructured interview with at least 6 participants from each health system (UCSF and SFDPH) for a minimum total of 12 and continue interviewing more participants (if needed) until we reach thematic saturation. Previous studies have suggested a small sample (*N*=10) can collect most of the salient ideas from semistructured interviews with basic elements for meta-themes emerging after just 6 interviews [15,16]. After we reach thematic saturation, we will stop conducting in-person interviews, but we will email Web links to the online exit survey to all remaining participants not invited for in-person interviews. The first part of the exit survey will collect data on clinician factors such as professional degree (MD vs nurse practitioner [NP]), years of practice, and medical specialty (family medicine vs internal medicine). We used a modified Technology Acceptance Model (TAM) framework to inform construction of the semistructured interview guide and the second part of the survey. TAM is a validated theory of technology acceptance that has been widely used outside of health care and has become an important theoretical tool for health IT research [17]. As a theory, TAM suggests perceived usefulness (PU) and perceived ease-of-use (PEU) as the 2 major factors that influence how users come to accept and use a technology. Our survey will also measure participants’ willingness or intention to use the collective intelligence platform beyond this study period—the interview guide will explore perceived barriers and facilitators that could influence their willingness to use. In addition to these concepts, our interview guide also includes additional constructs such as general satisfaction, trust, and system facilitators as well as supplemental open-ended questions to allow free expression of ideas. We will also ask participants to provide suggestions about the visual and content display of the platform output (see Multimedia Appendices 2 and 3 for contents of the exit survey and interview guide).

### Outcomes and Measurements

#### Primary and Secondary Outcomes

Our primary outcome will be self-reported diagnostic confidence as measured by response to the survey question in the postvisit questionnaire that asks participants after reviewing the collective intelligence output for cases randomized to the intervention group: “How confident do you feel about your diagnosis for this patient?” (Not at all, somewhat, moderately, and very). We chose this outcome based on previous literature in diagnostic safety suggesting that diagnostic confidence plays an important role in medical management, evolves over time in the process of making a diagnosis, and the ability to move diagnostic confidence may contribute to avoiding diagnostic error [18,19]. Furthermore, our preliminary studies for the proposed trial suggested that clinicians’ confidence in their clinical assessment is an important factor in their cognitive process for decision making and future approach to similar cases. We also chose this measure because it is likely the most sensitive to the impact of the collective intelligence on clinicians’ complex diagnostic decision-making process, whereas clinical outcomes such as *time to diagnosis* and diagnostic accuracy can be confounded by external health care system factors such as time to receive test results, financial incentives for diagnostic testing, and other factors. The secondary outcome will be *time to diagnosis*, defined as the interval between the time of the clinic visit from which a case with diagnostic uncertainty was identified for entry into the collective intelligence platform and the time at which the clinician will have established a diagnosis for that symptom or sign. We will ascertain the time of established diagnosis as the time at which the clinician documents the new diagnosis, the result for a confirmatory diagnostic testing becomes available, or empiric treatment was initiated. We will choose the earliest occurrence of any of these three events as the time of established diagnosis.

#### Exploratory Clinical Outcomes

We will measure and analyze specified, additional outcomes that are relevant to the diagnostic process. We will define diagnostic accuracy as having the correct diagnosis listed in the top 3 answers of the collective intelligence output (for intervention cases) or in the top 3 of the clinician’s list of possible diagnoses collected via survey data (for control cases). In addition, we will measure the number of diagnostic tests related to the initial complaint, ordered by the clinician within 30 and 90 days of the case presentation, and the number of unexpected visits (drop-in, urgent care, or emergency room) related to the initial presentation within 14 and 30 days of the index visit. Unexpected visits have been associated with higher risk of diagnostic error [1]. We refer to these as exploratory outcomes because we expect them to be highly variable both within and between clinicians and do not know whether we will have the statistical power to analyze them. Descriptive analyses of these outcomes may generate hypotheses for future study.

#### Satisfaction and Usability

We will use the exit survey and interview described above to assess the usability of collective intelligence to assist clinicians’ diagnostic process in routine primary care and urgent care cases and examine their willingness to use collective intelligence in practice. Through the survey, we will determine the Net Promoter Score (NPS) as a quantitative measure of user satisfaction and willingness to use and a modified TAM score (overall and for each theoretical variable) as a quantitative measure of usability. The NPS is based on a single question: “How likely is it that you would recommend our service to a friend or colleague?” This score is increasingly used in health services research as a summary of consumer satisfaction [20]. The theoretical variables comprising the TAM score include PU, PEU, Trust, perceived facilitators, and intention to use.

#### Electronic Health Record Chart Review for Ascertainment and Adjudication of Outcomes and Other Variables

A total of 2 study investigators will independently review the medical record 6 months after the initial presentation of the case to ascertain the final diagnosis and exclude cases with persistent diagnostic uncertainty after 6 months. We will resolve discrepancies in adjudication by consensus. Our chart review will also capture the number of comorbid conditions (based on the problem list and the 10th revision of the International Statistical Classification of Diseases billing codes entered by the clinician for the visit), the type of visit (new, returning, drop-in, or urgent care), the number of diagnostic tests related to the initial presentation ordered by the clinician within 30 and 90 days of the case presentation, and the number of unexpected visits related to the initial presentation (drop-in, urgent care, or emergency room) within 14 and 30 days of the index visit. We will use the same 2-investigator adjudication process to ascertain diagnostic tests and unexpected visits related to the initial presentation.

### Analysis Plan

#### Primary Outcome

We will perform a bivariate logistic regression with the intervention status (clinician access to collective intelligence output vs no access to collective intelligence) as the predictor and diagnostic confidence as the outcome, clustering on clinician. Self-reported diagnostic confidence will be treated as a categorical variable with “not at all” as the reference group.

#### Secondary Outcome

We will use Cox regression to compare time-to-diagnosis (T2Dx) between the intervention versus the control cases (ie, intervention status as primary predictor and T2Dx as outcome).

#### Survey Analysis

We will use *t-* test statistics to describe the NPS and TAM score (overall and for each variable) by clinician characteristic (NP vs MD; years of practice; and specialty—internal vs family medicine).

#### Exploratory Analyses

We will perform mixed-effect multivariable logistic regressions to compare diagnostic confidence, T2Dx, and diagnostic accuracy of intervention versus control cases, accounting for clustering by clinician and adjusted for baseline diagnostic uncertainty, perceived case difficulty, patient age, race, gender, number of comorbid conditions, type of visit (new, returning, drop-in, or urgent care), and primary care clinician characteristics such as professional degree (NP vs MD), specialty (family vs internal medicine), and years of experience. We have chosen covariates a-priori based on clinical judgment. For cases randomized to the intervention, mixed-effect bivariate logistic regressions will compare physicians’ diagnostic accuracy before and after reviewing the collective intelligence, adjusted for the aforementioned clinician characteristics. We will use bivariate and multivariable linear regressions to compare the number of diagnostic testing and use logistic regressions to compare the occurrence of unexpected clinical visits at 30 days between intervention versus control cases.

#### Multiple Hypothesis Testing

We will report the results of the hypothesis test for the primary outcome without adjustment for multiple hypothesis testing. No formal penalization for multiple hypothesis testing is planned for the secondary, subgroup, or exploratory outcome analyses, as we will treat them as exploratory and hypothesis generating. We will report 95% CIs for all point estimates.

#### Handling of Missing Data

Our general approach to missing data will be multiple imputation. It is possible that our primary outcome of diagnostic confidence will have a level of missing data, as it will depend on the response rate to survey questions. In addition to multiple imputation under the standard assumption that data are missing at random, given the covariates and outcomes that are observed, we will also implement sensitivity analyses using imputation under plausible missing-not-at-random scenarios. To maximize survey completion rates and thereby minimize the level of missing data, we will send 3 email reminders at 3 days, 7 days, and 14 days if the initial survey request was not completed. We will leave hard copies of the survey in clinicians’ mailboxes if the participants have not completed the online survey after the 3 email reminders.

#### Sample Size Justification

We planned our sample size using the primary outcome of clinician confidence in diagnosis, rated on a case-by-case basis. We anticipated an ability to detect (with 80% power) a 20% difference in clinicians rating their confidence in the case diagnosis as somewhat or very high, when comparing cases with versus without collective intelligence feedback. Assuming clustering of confidence ratings within clinicians at *r*=.3, we estimated that we need 33 clinicians with about 20 cases in total (10 with collective intelligence and 10 without collective intelligence; n=660 total cases).

#### Qualitative Analysis of Exit Interview

Qualitative analysis of interview transcripts will further examine barriers to usability and acceptance (ie, willingness to use) of the platform by clinicians in primary care clinical settings. Transcripts will be coded using an integrated inductive-deductive qualitative data analysis approach [21]. In particular, we will use the constant comparison method, an inductive qualitative data analysis approach in which data are broken down, compared for similarities and differences, and grouped together under similar conceptual themes [22] to uncover a wide variety of themes from the data, while also employing predetermined conceptual codes drawn from a modified TAM [23] to structure a deductive analysis of the data. A total of 2 study authors (KR and MH) will independently code the transcripts to identify preliminary themes through initial readings of the transcripts. Iterative discussions among all the study investigators will refine thematic categories and lead to a final set of salient themes identified across all the interviewees.

## Results

At the time of manuscript submission, the trial is actively enrolling participants, and the recruitment started on August 20, 2018. We have recruited 32 out of our recruitment goal of 33 participants at the San Francisco Health Network (SFHN) and UCSF Health primary care clinics. The majority of clinicians (25) work within the SFHN, and 7 are from UCSF Health primary care clinics. This study is funded until May 2020 and is approved by the UCSF Institutional Review Board (#17-23839) until January 2020. We have completed data collection as of June 2019 and will complete our proposed analysis by December 2019.

## Discussion

Diagnostic error is increasingly recognized as a significant public health concern. Extrapolations based on combining data from small studies suggest that approximately 12 million Americans experience a diagnostic error or delay every year in the ambulatory care setting [1]. However, to date, no prospective observational study of diagnosis in ambulatory care exists despite expert calls to address this important knowledge gap. Our study holds promise to close important gaps in the field of preventing diagnostic error in ambulatory care.

The study aims to improve diagnostic confidence in real-world ambulatory care settings using a collective intelligence technology platform that aims to assist primary care and urgent care clinicians in their diagnostic reasoning and decision making. Our robust mixed quantitative and qualitative analyses will help identify best use cases and clinical workflows for routine use of collective intelligence in ambulatory care. This work can then inform larger-scale work to estimate the impact of collective intelligence on diagnostic accuracy, and, ultimately, prevention of harm to patients. Findings will inform best practices to integrate digital health technology interventions for reducing diagnostic errors in primary care and urgent care clinics. This understanding can help with the adoption and tailoring of this and other collective intelligence platforms throughout safety-net health systems nationally.

## Appendix

Multimedia Appendix 1 Postvisit questionnaire.

Multimedia Appendix 2 Exit survey.

Multimedia Appendix 3 Interview guide for exit interview.

## Abbreviations

IT: information technology
MD: medical doctor
NP: nurse practitioner
NPS: Net Promoter Score
PEU: perceived ease-of-use
PU: perceived usefulness
SFDPH: San Francisco Department of Public Health
SFHN: San Francisco Health Network
T2Dx: time-to-diagnosis
TAM: Technology Acceptance Model
UCSF: University of California San Francisco

## References

[1] Singh H, Meyer AN, Thomas EJ (2014). The frequency of diagnostic errors in outpatient care: estimations from three large observational studies involving US adult populations. BMJ Qual Saf.

[2] Riches N, Panagioti M, Alam R, Cheraghi-Sohi S, Campbell S, Esmail A, Bower P (2016). The effectiveness of electronic differential diagnoses (DDX) generators: a systematic review and meta-analysis. PLoS One.

[3] Graber ML, Kissam S, Payne VL, Meyer AN, Sorensen A, Lenfestey N, Tant E, Henriksen K, Labresh K, Singh H (2012). Cognitive interventions to reduce diagnostic error: a narrative review. BMJ Qual Saf.

[4] Nurek M, Kostopoulou O, Delaney BC, Esmail A (2015). Reducing diagnostic errors in primary care. A systematic meta-review of computerized diagnostic decision support systems by the LINNEAUS collaboration on patient safety in primary care. Eur J Gen Pract.

[5] Chobanian AV, Bakris GL, Black HR, Cushman WC, Green LA, Izzo JL, Jones DW, Materson BJ, Oparil S, Wright JT, Roccella EJ, National Heart‚ Lung‚ and Blood Institute Joint National Committee on Prevention‚ Detection‚ Evaluation‚ and Treatment of High Blood Pressure, National High Blood Pressure Education Program Coordinating Committee (2003). The seventh report of the joint national committee on prevention, detection, evaluation, and treatment of high blood pressure: the JNC 7 report. J Am Med Assoc.

[6] Ball JR, Balogh E (2016). Improving diagnosis in health care: highlights of a report from the national academies of sciences, engineering, and medicine. Ann Intern Med.

[7] Woolley AW, Chabris CF, Pentland A, Hashmi N, Malone TW (2010). Evidence for a collective intelligence factor in the performance of human groups. Science.

[8] Sarkar U, Simchowitz B, Bonacum D, Strull W, Lopez A, Rotteau L, Shojania KG (2014). A qualitative analysis of physician perspectives on missed and delayed outpatient diagnosis: the focus on system-related factors. Jt Comm J Qual Patient Saf.

[9] Khare R, Good BM, Leaman R, Su AI, Lu Z (2016). Crowdsourcing in biomedicine: challenges and opportunities. Brief Bioinform.

[10] Kurvers RH, Krause J, Argenziano G, Zalaudek I, Wolf M (2015). Detection accuracy of collective intelligence assessments for skin cancer diagnosis. JAMA Dermatol.

[11] Wolf M, Krause J, Carney PA, Bogart A, Kurvers RH (2015). Collective intelligence meets medical decision-making: the collective outperforms the best radiologist. PLoS One.

[12] Sims MH, Bigham J, Kautz H, Halterman MW (2014). Crowdsourcing medical expertise in near real time. J Hosp Med.

[13] Abbasi J (2018). Shantanu Nundy, MD: the human diagnosis project. J Am Med Assoc.

[14] Boddupalli D, Nundy S, Bates D (2017). Collective Intelligence Outperforms Individual Physicians. Proceedings of the 39th Annual Meeting of the Society of Medical Decision Making.

[15] Weller SC, Vickers B, Bernard HR, Blackburn AM, Borgatti S, Gravlee CC, Johnson JC (2018). Open-ended interview questions and saturation. PLoS One.

[16] Guest G, Bunce A, Johnson L (2016). How many interviews are enough?: an experiment with data saturation and variability. Field Methods.

[17] Holden RJ, Karsh B (2010). The technology acceptance model: its past and its future in health care. J Biomed Inform.

[18] Ng CS, Palmer CR (2010). Analysis of diagnostic confidence: application to data from a prospective randomized controlled trial of CT for acute abdominal pain. Acta Radiol.

[19] Meyer AN, Payne VL, Meeks DW, Rao R, Singh H (2013). Physicians' diagnostic accuracy, confidence, and resource requests: a vignette study. JAMA Intern Med.

[20] Krol MW, de Boer D, Delnoij DM, Rademakers JJ (2015). The net promoter score--an asset to patient experience surveys?. Health Expect.

[21] Bradley EH, Curry LA, Devers KJ (2007). Qualitative data analysis for health services research: developing taxonomy, themes, and theory. Health Serv Res.

[22] Corbin J, Strauss A (2014). Basics of Qualitative Research: Techniques and Procedures for Developing Grounded Theory.

[23] Venkatesh V, Morris M, Davis GB, Davis FD (2003). User acceptance of information technology: toward a unified view. MIS Q.

