# Supplementary material for: Evaluation of a Health Information Technology–Enabled Collective Intelligence Platform to Improve Diagnosis in Primary Care and Urgent Care Settings: Protocol for a Pragmatic Randomized Controlled Trial
Source: JMIR Res Protoc. 2019 Aug 6;8(8):e13151. doi: 10.2196/13151 (PMC6701158; doi:10.2196/13151)
Supplement: Multimedia Appendix 1 [file resprot_v8i8e13151_app1.pdf]

Appendix 1: Post-visit questionnaire

Start of Block: Default Question Block

Please do not review Human Dx output until prompted.

Name

____

Date of clinic

____

Human Dx one-liner for this case

____
____
____
____
____

1. Please indicate your level of agreement with this statement:
The Human Dx one-liner accurately captures my clinical thinking for this case:

- ○ Strongly disagree
- ○ Disagree
- ○ Neither agree nor disagree
- ○ Agree
- ○ Strongly agree

1a. Comment on answer above

____

____

____

____

____

2. Would you have entered this case into Human Dx?

Human Dx is an online platform in which users can attempt to resolve clinical uncertainty by submitting cases for other physicians from around the world can comment and provide feedback on diagnostic assessment.

- ○ Yes
- ○ No

3. What three diagnoses are you considering for this case?

- ◯ Top Diagnosis ____
- ◯ 2nd Diagnosis ____
- ◯ 3rd Diagnosis ____

3a. Provide a description of diagnostic or treatment plan if desired (optional)

____

____

____

____

____

4. How difficult would you consider this clinical case?

- ◯ Not at all difficult
- ◯ Somewhat difficult
- ◯ Moderately difficult
- ◯ Very difficult

5. How uncertain do you feel about your diagnosis for this case?

- Not at all uncertain
- Somewhat uncertain
- Moderately uncertain
- Very uncertain

Display This Question:

If 5. How uncertain do you feel about your diagnosis for this case? != Not at all uncertain

6. For how long have you had diagnostic or treatment uncertainty for this case? (Please select one)

- This is a new issue
- Uncertainty for 3-6 months
- Uncertainty for 6-12 months
- More than 12 months

7. Were you given a Human Dx URL for this case?

- Yes
- No

Display This Question:

If 7. Were you given a Human Dx URL for this case? = No

8a. How confident do you feel about your diagnosis for this patient?

- ◯ Not at all confident
- ◯ Somewhat confident
- ◯ Moderately confident
- ◯ Very confident

Display This Question:

If 7. Were you given a Human Dx URL for this case? = Yes

Please review Human Dx output now.
8b. How confident do you feel about your diagnosis for this patient?

- ◯ Not at all confident
- ◯ Somewhat confident
- ◯ Moderately confident
- ◯ Very confident

Display This Question:

If 7. Were you given a Human Dx URL for this case? = Yes

9. After looking at the output, what three diagnoses are you considering for this case?

- ◯ Top Diagnosis ____
- ◯ 2nd Diagnosis ____
- ◯ 3rd Diagnosis ____

Display This Question:

If 7. Were you given a Human Dx URL for this case? = Yes

10. Has your treatment plan changed?

- ◯ Yes
- ◯ No

Display This Question:

If 7. Were you given a Human Dx URL for this case? = Yes

11. Has the collective opinion influenced your decision making for diagnostic work-up?

- ◯ Yes
- ◯ No

Display This Question:

If 10. Has your treatment plan changed? = Yes

Or 11. Has the collective opinion influenced your decision making for diagnostic work-up? = Yes

11a. Please elaborate on changes made in one sentence

____

Display This Question:

If 7. Were you given a Human Dx URL for this case? = Yes

12. How helpful did you find the collective opinion?

- ◯ Not at all helpful
- ◯ Somewhat helpful
- ◯ Moderately helpful
- ◯ Very helpful

Display This Question:

If 7. Were you given a Human Dx URL for this case? = Yes

13. Was this output delivered with enough time to influence patient care?

- ◯ Yes - I received this with enough time to influence me
- ◯ Yes - but I wanted it sooner
- ◯ No - I needed it sooner
- ◯ No - I would not be influenced regardless

End of Block: Default Question Block
