# Supplementary material for: Evaluation of a Health Information Technology–Enabled Collective Intelligence Platform to Improve Diagnosis in Primary Care and Urgent Care Settings: Protocol for a Pragmatic Randomized Controlled Trial
Source: JMIR Res Protoc. 2019 Aug 6;8(8):e13151. doi: 10.2196/13151 (PMC6701158; doi:10.2196/13151)
Supplement: Multimedia Appendix 2 [file resprot_v8i8e13151_app2.pdf]

Appendix 2: Exit Survey

Name: ________________________________

1. Demographic Information:

What is your age range?	☐ 20-29<br>☐ 30-39<br>☐ 40-49	☐ 50-59<br>☐ 60 and Over
Are you of Hispanic, Latino/a, or Spanish origin?	☐ Yes	☐ No
What is your racial background? (Select all that apply)	☐ American Indian or Alaska Native<br>☐ Asian<br>☐ Black or African American	☐ Native Hawaiian or Other Pacific Islander<br>☐ White<br>☐ Other
What is your gender?	☐ Female<br>☐ Male<br>☐ Transfemale	☐ Transmale<br>☐ Other

**2. For how many years have you been in practice?**	

**3. Indicate your training/specialty**	☐ MD – Internal Medicine<br>☐ MD – Family Medicine<br>☐ MD – other	☐ Physician's Assistant<br>☐ Nurse Practitioner

4. Considering your complete experience, how likely would you be to recommend Human Dx to a colleague or friend?

◯ 0 ◯ 1 ◯ 2 ◯ 3 ◯ 4 ◯ 5 ◯ 6 ◯ 7 ◯ 8 ◯ 9 ◯ 10

5. Please answer the following questions

Attitude

Using collective intelligence technology in patient care and management is a good idea	Using collective intelligence technology in patient care and management is unpleasant
◯ Strongly agree<br>◯ Agree<br>◯ Somewhat agree<br>◯ Neither agree nor disagree<br>◯ Somewhat disagree<br>◯ Disagree<br>◯ Strongly disagree	◯ Strongly agree<br>◯ Agree<br>◯ Somewhat agree<br>◯ Neither agree nor disagree<br>◯ Somewhat disagree<br>◯ Disagree<br>◯ Strongly disagree
Using collective intelligence technology is beneficial to my patient care and management<br>◯ Strongly agree<br>◯ Agree<br>◯ Somewhat agree<br>◯ Neither agree nor disagree<br>◯ Somewhat disagree<br>◯ Disagree<br>◯ Strongly disagree	

Perceived usefulness

Using collective intelligence technology cannot improve my patient care and management	Using collective intelligence technology cannot enhance my effectiveness in patient care and management
◯ Strongly agree<br>◯ Agree<br>◯ Somewhat agree<br>◯ Neither agree nor disagree<br>◯ Somewhat disagree<br>◯ Disagree<br>◯ Strongly disagree	◯ Strongly agree<br>◯ Agree<br>◯ Somewhat agree<br>◯ Neither agree nor disagree<br>◯ Somewhat disagree<br>◯ Disagree<br>◯ Strongly disagree

Using collective intelligence technology can make my patient care and management easier

◯ Strongly agree
◯ Agree
◯ Somewhat agree
◯ Neither agree nor disagree
◯ Somewhat disagree
◯ Disagree
◯ Strongly disagree

I would find collective intelligence technology not useful for my patient care and management

◯ Strongly agree
◯ Agree
◯ Somewhat agree
◯ Neither agree nor disagree
◯ Somewhat disagree
◯ Disagree
◯ Strongly disagree

Perceived ease of use

Learning to operate collective intelligence technology would not be easy for me

◯ Strongly agree
◯ Agree
◯ Somewhat agree
◯ Neither agree nor disagree
◯ Somewhat disagree
◯ Disagree
◯ Strongly disagree

Using collective intelligence technology cannot enhance my effectiveness in patient care and management

◯ Strongly agree
◯ Agree
◯ Somewhat agree
◯ Neither agree nor disagree
◯ Somewhat disagree
◯ Disagree
◯ Strongly disagree

Using collective intelligence technology can make my patient care and management easier

◯ Strongly agree
◯ Agree
◯ Somewhat agree
◯ Neither agree nor disagree
◯ Somewhat disagree
◯ Disagree
◯ Strongly disagree

I would find collective intelligence technology not useful for my patient care and management

◯ Strongly agree
◯ Agree
◯ Somewhat agree
◯ Neither agree nor disagree
◯ Somewhat disagree
◯ Disagree
◯ Strongly disagree

Behavioral intention

I intend to use collective intelligence technology for patient care as often as needed

◯ Strongly agree
◯ Agree
◯ Somewhat agree
◯ Neither agree nor disagree
◯ Somewhat disagree
◯ Disagree

Whenever possible, I intend not to use collective intelligence technology for patient care

◯ Strongly agree
◯ Agree
◯ Somewhat agree
◯ Neither agree nor disagree
◯ Somewhat disagree
◯ Disagree

◯ Strongly disagree	◯ Strongly disagree

To the extent possible, I would use collective intelligence technology in my patient care frequently

◯ Strongly agree
◯ Agree
◯ Somewhat agree
◯ Neither agree nor disagree
◯ Somewhat disagree
◯ Disagree
◯ Strongly disagree
