# Supplementary material for: Evaluation of a Health Information Technology–Enabled Collective Intelligence Platform to Improve Diagnosis in Primary Care and Urgent Care Settings: Protocol for a Pragmatic Randomized Controlled Trial
Source: JMIR Res Protoc. 2019 Aug 6;8(8):e13151. doi: 10.2196/13151 (PMC6701158; doi:10.2196/13151)
Supplement: Multimedia Appendix 3 [file resprot_v8i8e13151_app3.pdf]

Appendix 3: Interview guide for exit interview

Demonstration Study Exit Interview Flow Checklist:

- Facilitator(s) to print out Human Dx output or cases to review and reimbursement certification forms
- Participant to fill out reimbursement info.
- Begin audio recording
- Facilitator give overview of session and disclaimer

Overview (60 minute interview):

1. Ask general questions on the seven domains:
    a. Uncertainty/Types of Cases
    b. Trust (including quality/accuracy and people reviewing cases)
    c. Understanding/Treatment Planning
    d. Confidence
    e. Workflow/Timing and Next Steps
    f. Ease of use
    g. Usefulness
2. Review Human Dx Output #1 for a case with higher confidence
3. Review Human Dx Output #2 for a case with lower confidence
4. Wrap up and thank participant for their time

FACILITATOR: GENERAL QUALITATIVE QUESTIONS

(Verbal) *Now I am going to ask you some open-ended questions about your experience with Human Dx.*

1) **Uncertainty/Types of Cases: Did you feel that the cases we entered on the platform and generated collective intelligence for were appropriate for consultation using the platform? Why or why not?**

If you had entered cases on your own, would you have chosen to seek consultation on the same cases we entered?

i. Which (specific or types of) cases would you not seek consultation on and why?

For what reasons would you think to enter a case in Human Dx?
Probe: Describe the kinds of cases you would enter on the platform.

What would you be looking for from the Human Dx output? What kind of help or feedback would you want ideally?

2) **Trust:** Overall did you <u>trust</u> the collective intelligence generated from Human Dx?
    i. What made you trust or distrust the app? (e.g. total number of responses, number of responses for a given diagnosis, rationales)

Did you think the output was accurate?

i. What aspects contributed to your belief in the app's accuracy (e.g. size of collective opinion, reasonable diagnoses and rationales)?

Tell me about how the <u>background of respondents</u> affected your trust of the collective opinion.

i. Did the institution, specialty (FM vs. IM) affect your trust?

Is there anything about the app that could be changed to make you trust the collective opinion more?

3) Understanding/Treatment planning: Can you tell me about how the collective opinion influenced your understanding or thinking process for a given clinical presentation?

Tell me about how the collective opinion influenced your diagnostic decision-making.

- **i.** Why or why not?

Tell me about how the collective opinion influenced your treatment decision-making.

i. Why or why not?

What are potential harm and disadvantages to using collective intelligence?
PROBE: How concerning are these potential harms? What factors can you think of to mitigate the potential harm?

4) **Confidence:** Tell me about how the collective opinion influenced your confidence in your assessment and plan?

ii. Did the collective opinion make you more confident or more confused? Why?

If the output lowered your confidence, do you think this made you more or less likely to use the platform in the future? Why?

If you had to use collective intelligence in your clinical practice, is the timing of the output appropriate for your workflow and next steps? Probe: What would the ideal timing of the output be for you?

What about this platform, your current practice setting, or just your overall sentiment would make you less willing or more likely to use it?

5) **Ease of use:** Was the output easy to understand/view and navigate?
   i. What would make it easier to use?

What problems did you experience when reviewing the collective intelligence output?

i. Where did you get frustrated?

Does the output contain all the relevant information?

Could any information be combined or altered to improve usefulness/relevance?

How would you change the output if you could? Please feel free to comment on both the content and display/design?

6) **Usefulness:** Tell me about how <u>useful</u> you found the peer-to-peer consultation for your cases.
    i. In what aspects did you find the app useful? In what ways was it not useful?

How do you interpret helpfulness or usefulness?

7) **Facilitators (or facilitation conditions):** Does your institution have the necessary organizational and/or technical infrastructure to support your use of a collective intelligence platform?

    a. **Probe:** What types of structural changes would you recommend to facilitate use of this platform?

8) **Workflow:** Can you imagine integrating use of the platform into your <u>current workflow</u>? How? **Probe: Would it be easy or difficult?**

    a. **Workflow for receiving and reviewing the collective intel. What if you didn't have to enter the cases into the platform yourself?**

b. **Workflow for entering cases and receiving/reviewing collective intel. Can you imagine a workflow where you or your staff input cases into the platform**

FACILITATOR: HUMAN DX CASE #1 FOR HIGHER CONFIDENCE

(Verbal) *Now I am going to ask you to walk through your thought process on a specific case. (Show participant already printed out Human Dx output)*

Reference survey responses when asking questions.

On this case, your confidence higher. Tell me more about that.

What are your thoughts on the platform even though your confidence higher?

How important do you feel that a change in confidence is to the app's usefulness?

Did you learn something from the Human Dx output?

When filling out our follow up survey on this case, how did you interpret question #3 about your top three diagnoses?

- PROBE: How did you decide which diagnoses to list? (eg. most likely diagnosis, highest acuity)

FACILITATOR: HUMAN DX CASE #2 FOR LOWER CONFIDENCE

(Verbal) *Now I am going to ask you to walk through your thought process on a specific case. (Show participant already printed out Human Dx output)*

Reference survey responses when asking questions.

On this case, your confidence lower. Tell me more about that.

What are your thoughts on the platform even though your confidence lower?

How important do you feel that a change in confidence is to the app's usefulness?

Did you learn something from the Human Dx output?

When filling out our follow up survey on this case, how did you interpret question #3 about your top three diagnoses?

- PROBE: How did you decide which diagnoses to list? (eg. most likely diagnosis, highest acuity)

What are your thoughts overall? How would you summarize your opinion about the platform and the collective intelligence output?
